# 1003. Case Study. Bridging Catastrophic 90% TBSA Burn to Autograft Using a Synthetic Electrospun Fiber Matrix (SEFM)

**DOI:** 10.1093/jbcr/irag033.191

**Published:** 2026-04-06

**Authors:** Ryu Tran, Brianna Taylor, Madison Landers, Kevin Dare

**Affiliations:** University of Oklahoma-Tulsa School of Community Medicine, Tulsa, OK; University of Oklahoma-Tulsa School of Community Medicine, Tulsa, OK; University of Oklahoma-Tulsa School of Community Medicine, Tulsa, OK; Alexander Burn Center, Tulsa, OK

## Abstract

**Patient Presentation (age range, injury details, relevant history):**

An 18-year-old male sustained catastrophic 90% total body surface area (TBSA) burns in a warehouse explosion. The injury encompassed trunk, all extremities, and buttocks, with only limited donor sites available for autografting. Patient was otherwise healthy without significant past medical history.

**Clinical Challenges:**

Severe burns of this scale carry extremely poor survival, with high risk of infection, limited donor site availability, and the inherent fragility and cost of biologic dressings. Achieving durable temporary coverage capable of maintaining a viable wound bed until donor sites could be re-harvested was a central challenge. The buttock and perineal regions posed particular difficulty due to recurrent contamination and shear forces, historically resulting in high graft failure rates.

**Management Approach:**

After staged tangential excision, a synthetic electrospun fiber matrix (SEFM) was applied across 15 940 cm^2^ of wound surface. On post-burn day (PBD) 6, SEFM was placed on the back, buttocks, and posterior legs; on PBD 8, the arms, flanks, and anterior legs; and on PBD 13, the distal extremities. Wounds were dressed with silver-based interfaces. Definitive coverage began on PBD 27 using split-thickness autografts supplemented with epidermal suspension autografting to the arms, followed by staged re-harvesting of donor sites for lower extremity and residual arm coverage.

**Outcomes:**

SEFM rapidly integrated, resisted enzymatic degradation, and promoted robust granulation across 15 940 cm^2^. No infections occurred in SEFM-treated areas until after the matrix had granulated over. Definitive grafting commenced 19-21 days after SEFM placement, demonstrating non-inferior timelines comparable to existing bridging methods, with excellent graft take throughout. Even in the high-risk buttock region, SEFM preserved a healthy wound bed, though grafting of the buttocks was deliberately deferred to avoid donor site waste should grafting fail.

**Lessons Learned:**

SEFM enabled one of the largest reported applications of a synthetic electrospun fiber matrix in burn care, providing durable, infection-resistant coverage on a catastrophic scale. It supported donor site cycling, maintained wound bed quality, and demonstrated resilience even in contaminated, high-shear regions, allowing safe bridging to autograft.

**Applicability to Practice:**

This is, to our knowledge, the first report of SEFM successfully bridging to definitive autografting in a 90% TBSA burn. Its synthetic, fully resorbable structure and infection-resistant properties allowed durable coverage on a massive scale, highlighting SEFM’s potential to shift practice in the management of catastrophic burns with limited donor availability.

